# Association of dietary live microbe intake with abdominal aortic calcification in US adults: a cross-sectional study of NHANES 2013–2014

**DOI:** 10.3389/fnut.2023.1267607

**Published:** 2023-11-24

**Authors:** Xingwei Huo, Shanshan Jia, Xin Zhang, Lirong Sun, Xueting Liu, Lu Liu, Xianghao Zuo, Xiaoping Chen

**Affiliations:** ^1^Department of Cardiology, West China Hospital, Sichuan University, Chengdu, Sichuan, China; ^2^Second Department of Internal Medicine, Affiliated Hospital of Tibet University for Nationalities, Xianyang, Shaanxi, China

**Keywords:** dietary live microbes, abdominal aortic calcification, food-gut-health axis, NHANES, cross-sectional study

## Abstract

**Object:**

To explore the potential association between dietary live microbe intake and abdominal aortic calcification (AAC).

**Methods:**

We conducted a cross-section study based on the National Health and Nutrition Examination Survey (NHANES). We categorized the participants into three groups (low, medium, and high dietary intake of live microbes) according to Sanders’s dietary live microbe classification system and participants’ 24-h dietary recall data. AAC was quantified by using dual-energy X-ray absorptiometry (DXA) and diagnosed by using the Kauppila AAC-24 score system. The analyses utilized weighted logistic regression and weighted linear regression.

**Results:**

A total of 2,586 participants were included. After the full adjustment for covariates, compared to participants with a low dietary live microbe intake, participants with a high dietary live microbe intake had a significantly lower risk of severe AAC (OR: 0.39, 95% CI: 0.22, 0.68, *p* = 0.003), and the AAC score was also significantly decreased (β:−0.53, 95% CI: −0.83, −0.23, *p* = 0.002).

**Conclusion:**

In this study, more dietary live microbial intake was associated with lower AAC scores and a lower risk of severe AAC. However, more research is needed to verify this.

## 1 Introduction

Vascular calcification (VC) is the abnormal deposition of calcium and phosphorus in the walls of blood vessels (1). Although studies on VC have primarily focused on coronary artery calcification (CAC), the multi-ethnic study of atherosclerosis (MESA) study suggests that abdominal aortic calcification (AAC) occurs earlier and is a more effective prognostic marker than CAC, independent of CAC scores (2, 3). Current studies have established a strong correlation between AAC and cardiovascular events as well as mortality (3–5), but another study still believed AAC is an underestimated cardiovascular disease risk factor (6). As the AAC severity increases, the risk of fatal cardiovascular events and mortality also increases significantly (7, 8).

Since the progression of VC is difficult to reverse, prevention and treatment are extremely important (9). Although VC is often considered the advanced stage of atherosclerosis (AS) (3), current medications targeting AS have shown limited efficacy in treating VC (10). Several drugs, such as bisphosphonates, calcium channel blockers, vitamin K, and dietary magnesium, may have therapeutic potential, but there is still a lack of robust clinical evidence (3, 11, 12). To date, there are no approved treatment strategies for preventing or managing AAC (13).

Adopting healthy diets is widely recognized as an effective approach to preventing cardiovascular diseases (CVD) (14). Healthy diets such as anti-inflammatory diets (15), Mediterranean diets (14), and DASH diets (16), decreased the risk of having AAC. These studies mainly focused on the role of food ingredients but ignored the impact of dietary live microbes. Studies have found that diets can influence gut flora composition and metabolites (17) and impact disease susceptibility (18, 19). Therefore, there is growing interest in the health effects of live microbes in diets. Recently, Sanders et al. (20) used the National Health and Nutrition Examination Survey (NHANES) database to assess the number of live mircrobes in various foods. They also discovered live microbe-rich diets improved health outcomes, including lower BMI, blood pressure, lipids, glucose, insulin, and inflammation level (21). These benefits from dietary live microbes may prevent diseases. Based on NHANES data and Sanders’ dietary live microbe classification system, we explored the association between dietary live microbes and AAC.

## 2 Materials and methods

### 2.1 Data source and participants

**FIGURE 1:**
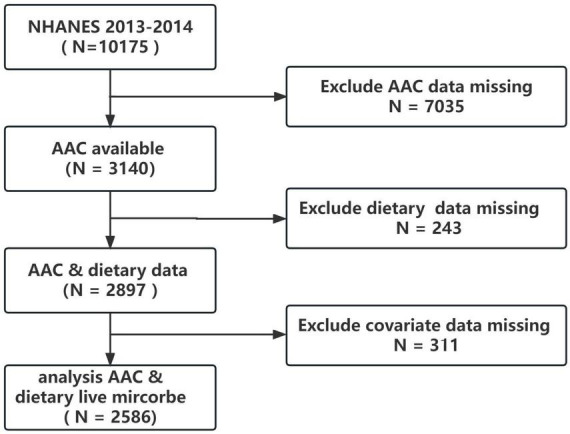
Participant flowchart.

The NHANES survey design follows a complex, multistage probability sampling method to obtain nationally representative samples of the non-institutionalized US civilian population. The data collected includes demographics, physical examinations, laboratory tests, dietary information, and other questionnaires. The National Center for Health Statistics (NCHS) Institutional Review Board has approved the NHANES protocol, and participants provided informed consent before the collection of personal information, blood, and urine samples. The 2013–2014 cycle was chosen because it was the only cycle that dual X-ray absorptiometry (DXA) scans for AAC were performed. All data in this study is publicly available on the NHANES website.^1^ For the 2013–2014 cycle, the total population was 10,175. However, only participants over 40 years old received the DXA examination to obtain AAC score data. After excluding those with missing AAC data (*n* = 7035), missing dietary data (*n* = 243), and missing covariate data (*n* = 311), the final analysis consisted of 2586 participants, as depicted in Figure 1.

### 2.2 Dietary live microbe intake category

The dietary live microbe intake was estimated by using 24-h dietary recall data from NHANES. The food codes in the NHANES database were linked to the United States Department of Agriculture (USDA) to obtain the food composition and energy content data. A team of four experts, relying on values reported in the primary literature, estimated the levels of live microbes (CFU/g) for 9,388 food codes across 48 subgroups in the NHANES database. The experts categorized microbial levels as low (< 10^4^ CFU/g), medium (10^4^–10^7^ CFU/g), or high (> 10^7^ CFU/g) based on the quantity of live microorganisms per gram of food. Any uncertain or conflicting data was resolved through external consultation. In short, the low class is mainly pasteurized foods, the medium class is mainly fresh fruits and vegetables that have not been peeled and the high class is fermented foods and probiotic supplements that have not been pasteurized. Although this classification method is useful for estimating liver microbes in foods, it may not be suitable for estimating entire diet live microbe intake. Following the approach of Sanders et al. (20), participants were grouped into three categories according to their overall live microbe intake from all foods: (1) low diet microbe intake group (only low level foods), (2) medium diet microbe intake group (medium level but not high level foods), and (3) high diet microbe intake group (any high level foods). This previously validated method allowed for the classification of participants’ diets based on estimated live microbe content (22, 23).

### 2.3 Outcome

The outcome variable in this analysis was AAC. The NHANES Mobile Examination Center (MEC) conducted lateral DXA scans of the thoracic and lumbar spine in 2013–2014. Exclusion criteria included age under 40 years, pregnancy, weight over 450 pounds, or recent barium contrast use in the past 7 days. The Kauppila score system (24) was utilized to evaluate AAC from the DXA scans. This involved dividing the anterior and posterior aortic walls corresponding to vertebral levels L1–L4 into 8 segments. Each segment was scored from 0 to 3 according to the extent of calcific deposits visualized. The sum of the 8 segment scores produced the total AAC score, ranging from 0 to 24. We defined AAC presence as a score above 0 and severe AAC as a score above 6. In addition, we performed sensitivity analysis using the AAC-8 score (Schousboe score), with a total score ranging from 0 to 8 (25). A score of 3 or more is defined as severe AAC (26).

### 2.4 Covariates

To avoid the influence of confounding factors, covariates were included in the analysis based on known or potential relationships with AAC. Demographic factors consisted of age, gender, race, education, body mass index (BMI), systolic blood pressure (SBP), diastolic blood pressure (DBP), smoking, and alcohol use. Medical conditions included hypertension, diabetes, and congestive heart failure. Laboratory measurements collected were serum calcium, phosphorus, total 25-hydroxyvitamin D, potassium, HbA1c, uric acid, creatinine, total cholesterol, white blood cells (WBC), and estimated glomerular filtration rate (eGFR). Use of antidiabetic, antihyperlipidemic, and antihypertensive medications was recorded. Dietary energy was also considered a covariate. Smoking was defined as having smoked ≥ 100 cigarettes over their lifetime. The diagnostic criteria for diabetes included self-reported diabetes, HbA1c ≥ 6.5%, fasting serum glucose ≥ 126 mg/dL, random serum glucose > 11.1 mmol, or 2-h postprandial glucose ≥ 200 mg/dL, or any self-reported insulin and antidiabetic medication use. Hypertension was defined as having a history of hypertension, an average SBP ≥ 140 mmHg, or DBP ≥ 90 mmHg based on at least three standard consecutive seated measurements or self-reported use of any hypertension-related medications. The eGFR was calculated by using the Chronic Kidney Disease Epidemiology Collaboration (CKD-EPI) creatinine equation (27).

### 2.5 Statistical analyses

All analyses accounted for the complex, multistage probability sampling design of NHANES by incorporating appropriate sampling weight. The R (Core Team, Vienna, Austria, version 4.1.2) and the survey package were utilized for complex sampling analysis. Continuous variables were reported as weighted means with standard errors (SE) while categorical variables were reported as weighted proportions. Baseline clinical characteristics were compared among groups using weighted *t*-tests and Rao-Scott chi-square tests. The association between dietary live microbe intake and AAC score was analyzed using weighted linear regression. Weighted logistic regression examined the association between dietary live microbe intake and severe AAC. Three regression models were constructed: model 1, adjusted for non-covariates; model 2, adjusted for age, gender, race, and education; and model 3, further adjusted for BMI, smoking, alcohol use, SBP, DBP, uric acid, creatinine, eGFR, HbA1c, total cholesterol, potassium, WBC, dietary energy, medical conditions (diabetes, hypertension, and congestive heart failure), medication use (antihypertensive, antihyperlipidemic, and antidiabetic), and bone metabolism markers (serum calcium, phosphorus, and total 25-hydroxyvitamin D). To avoid bias caused by deleting samples with missing covariates, we performed multiple imputations as a sensitivity analysis. In addition, we tested the robustness of the association using the AAC-8 score system. Subgroup analyses were conducted to examine potential effect modification by stratifying weighted multivariate regression models for age, gender, education, BMI, hypertension, diabetes, eGFR, smoking, and alcohol use. Trend tests to detect potential dose-response effects. A two-sided *P*-value < 0.05 was considered statistically significant.

## 3 Results

### 3.1 Clinical characteristics of participants

Table 1 presented the clinical characteristics of 2,586 individuals categorized by different dietary live microbe groups. The average age of the study population was 57.71 ± 0.29 years with a gender distribution of 51.75% females and 48.25% males. The average AAC scores for the overall population were 1.42 ± 0.10. Notably, there was a significant difference in the mean AAC scores among the three groups, with the high dietary live microbe intake group scoring the lowest, followed by the medium intake group. Furthermore, there were notable distinctions in several demographic and clinical parameters including age, gender, race, education, BMI, DBP, smoking, creatinine, HbA1c, total 25-hydroxyvitamin D, energy intake, and the prevalence of hypertension and diabetes among three groups (all *P* < 0.05). However, there were no statistically significant differences observed for SBP, alcohol use, uric acid, total cholesterol, WBC, eGFR, phosphorus, calcium, potassium, drug use, and the prevalence of congestive heart failure among the three groups.

**TABLE 1:** Clinical characteristics of participants by dietary live microbe groups.

Variables	Total	Low	Medium	High	*P*-value
Age (years)	57.71 (0.29)	56.40 (0.49)	58.59 (0.65)	58.00 (0.41)	0.007
Gender (%)					0.004
Female	51.75 (0.03)	45.45 (2.04)	54.35 (1.52)	55.12 (2.13)	
Male	48.25 (0.03)	54.55 (2.04)	45.65 (1.52)	44.88 (2.13)	
Race (%)					< 0.001
Black	9.62 (0.01)	14.48 (2.48)	9.02 (1.27)	5.35 (0.94)	
Mexican	6.84 (0.02)	5.97 (1.35)	9.48 (2.46)	4.60 (1.33)	
Other	12.10 (0.01)	10.85 (1.74)	16.38 (1.46)	8.32 (1.57)	
White	71.45 (0.07)	68.70 (4.04)	65.13 (3.60)	81.74 (2.87)	
Education level (%)					< 0.001
Less than high school	14.45 (0.02)	21.25 (3.14)	13.76 (1.69)	8.32 (1.45)	
High school or GED	21.71 (0.02)	26.59 (2.25)	20.96 (2.47)	17.60 (1.85)	
Above high school	63.83 (0.05)	52.16 (3.94)	65.28 (3.43)	74.08 (2.20)	
BMI (kg/m^2^)	28.48 (0.18)	29.23 (0.41)	28.23 (0.26)	28.00 (0.22)	0.035
SBP (mmHg)	125.36 (0.65)	126.03 (1.29)	126.14 (0.65)	123.76 (1.01)	0.085
DBP (mmHg)	71.19 (0.37)	72.09 (0.58)	70.32 (0.48)	71.31 (0.70)	0.047
Smoking (%)	45.90 (0.04)	53.86 (2.69)	44.48 (2.29)	39.42 (2.63)	< 0.001
Alcohol use (%)	89.51 (0.06)	90.62 (0.81)	88.12 (1.85)	90.03 (2.38)	0.381
Hypertension (%)	51.27 (0.03)	54.53 (1.83)	53.51 (1.81)	45.27 (3.40)	0.039
Diabetes (%)	18.10 (0.01)	20.31 (1.58)	19.13 (1.86)	14.62 (1.04)	0.031
Congestive heart failure (%)	2.70 (0.00)	3.10 (0.55)	2.86 (0.64)	2.09 (0.61)	0.531
Antidiabetic (%)	11.39 (0.01)	14.26 (1.64)	10.58 (1.00)	9.40 (1.29)	0.069
Antihypertensive (%)	39.15 (0.02)	41.04 (2.68)	40.20 (2.01)	35.98 (2.26)	0.310
Antihyperlipidemic (%)	30.34 (0.02)	29.44 (1.85)	30.51 (1.86)	31.06 (3.04)	0.845
Serum uric acid (μmol/L)	321.10 (1.73)	330.53 (5.27)	319.01 (3.55)	313.92 (2.53)	0.097
HbA1c (%)	5.76 (0.03)	5.82 (0.05)	5.77 (0.04)	5.67 (0.04)	0.019
Total cholesterol (mmol/L)	5.07 (0.02)	5.02 (0.04)	5.04 (0.04)	5.14 (0.05)	0.237
WBC, (1,000 cells/uL)	7.19 (0.06)	7.36 (0.08)	7.19 (0.09)	7.00 (0.12)	0.101
eGFR ml⋅ (min × 1.73 m^2^)^–1^	84.09 (0.43)	84.87 (0.56)	83.70 (0.89)	83.77 (0.99)	0.337
Serum creatinine (mmol/l)	81.46 (0.79)	82.89 (0.77)	81.84 (1.31)	79.53 (1.00)	0.006
Total 25-hydroxyvitamin D (nmol/L)	75.61 (1.47)	69.56 (1.61)	77.25 (1.77)	79.86 (1.63)	< 0.001
Serum calcium (mmol/l)	2.37 (0.00)	2.36 (0.01)	2.37 (0.00)	2.37 (0.01)	0.423
Serum phosphorus (mmol/l)	1.23 (0.01)	1.22 (0.01)	1.24 (0.01)	1.24 (0.01)	0.077
Serum potassium (mmol/l)	4.03 (0.02)	4.02 (0.02)	4.03 (0.02)	4.04 (0.03)	0.649
Energy (kcal)	2,052.11 (24.98)	2,025.40 (37.13)	1,987.05 (28.71)	2,156.42 (55.11)	0.031
AAC24 score	1.42 (0.10)	1.61 (0.11)	1.54 (0.16)	1.09 (0.13)	0.030
Sever AAC (%)					0.037
No	92.36 (0.06)	90.65 (0.73)	91.54 (1.24)	95.07 (1.12)	
Yes	7.64 (0.01)	9.35 (0.73)	8.46 (1.24)	4.93 (1.12)	

GED, general educational development; BMI, body mass index; SBP, systolic blood pressure; DBP, diastolic blood pressure; eGFR, estimated glomerular filtration rate; WBC, white blood cells; AAC, abdominal aortic calcification.

### 3.2 Association between dietary live microbes and AAC

The relationship between dietary live microbes and AAC was evaluated by employing weighted multivariate linear regression and weighted multivariate logistic regression. Three models were constructed and the results are presented in Table 2. Compared to individuals with low dietary live microbe intake group, those in high dietary live microbe intake group exhibited significantly lower AAC scores (model 1: β = −0.52, 95% CI: −0.90, −0.15, *p* = 0.009; model 2: β = −0.68, 95% CI: −1.05, −0.31, *p* = 0.004; model 3: β = −0.53, 95% CI: −0.83, −0.23, *p* = 0.002). Furthermore, after accounting for covariates, the medium dietary live microbe intake group also demonstrated lower AAC scores than the low intake group (model 1: β = −0.07, 95% CI: −0.34, 0.19, *p* = 0.557; model 2: β = −0.30, 95% CI: −0.49, −0.12, *p* = 0.007; model 3: β = −0.29, 95% CI: −0.50, −0.08, *p* = 0.011).

**TABLE 2:** Association between dietary live microbes and AAC.

	β ^a^/OR^b^ (95% CI^c^), *p*-value			
	**Low**	**Medium**	**High**	***p* for trend**
**AAC^d^ score**				
Model 1^e^	Reference	−0.07 (−0.34, 0.19) 0.557	−0.52 (−0.90, −0.15) 0.009	0.009
Model 2^f^	Reference	−0.30 (−0.49, −0.12) 0.007	−0.68 (−1.05, −0.31) 0.004	0.003
Model 3^g^	Reference	−0.29 (−0.50, −0.08) 0.011	−0.53 (−0.83, −0.23) 0.002	0.002
**severe AAC^d^**				
Model 1^e^	Reference	0.90 (0.69, 1.16) 0.372	0.50 (0.29, 0.88) 0.020	0.008
Model 2^f^	Reference	0.61 (0.46, 0.80) 0.004	0.38 (0.21, 0.68) 0.007	0.003
Model 3^g^	Reference	0.58 (0.43, 0.77) 0.001	0.39 (0.22, 0.68) 0.003	0.002

^a^β, effect sizes.

*^b^*OR, odds ratio.

*^c^*95% CI, 95% confidence interval.

*^d^*AAC, abdominal aortic calcification.

*^e^*Model 1: adjusted for non-covariates.

*^f^*Model 2: adjusted for age, gender, race, education.

*^g^*Model 3: further adjusted for body mass index, systolic blood pressure, diastolic blood pressure, smoking, alcohol use, hypertension, diabetes, congestive heart failure, HbA1c, total cholesterol, uric acid, estimated glomerular filtration rate, creatine, potassium, calcium, phosphorus, total 25-hydroxyvitamin D, white blood cells, antidiabetic, antihypertensive, antihyperlipidemic and dietary energy.

Additionally, for severe AAC, the results were similar. Compared to individuals in low dietary live microbe intake group, those in high dietary live microbe intake group exhibited lower risk of severe AAC (model 1, OR = 0.50, 95% CI: 0.29, 0.88, *p* = 0.020; model 2, OR = 0.38, 95% CI: 0.21, 0.68, *p* = 0.007; model 3, OR = 0.39, 95% CI: 0.22, 0.68, *p* = 0.003). Similarly, the medium dietary live microbe intake group also showed diminished risk (model 1: OR = 0.90, 95% CI: 0.69, 1.16, *p* = 0.372; model 2: OR = 0.61, 95% CI: 0.46, 0.80, *p* = 0.004; model 3: OR = 0.58, 95% CI: 0.43, 0.77, *p* = 0.001). In summary, more dietary live microbe intake was associated with lower AAC scores and lower risk of severe AAC.

### 3.3 Subgroup analysis

To examine the stability of the association between AAC and dietary live microbes, we conducted subgroup analyses, interaction tests, and trend tests between dietary live microbes and AAC across age, gender, education, hypertension, diabetes, eGFR, BMI, smoking, and alcohol use. Compared to those with low dietary live microbe intake, those who were older than 60 years, with hypertension, diabetes, eGFR ≥60ml ⋅ (min × 1.73 m^2^)^−1^, BMI greater than 25, smoking, or drinking were more likely to benefit from more dietary intake of live microbes. Only the interaction test for hypertension and BMI were meaningful. Interestingly, males benefited from medium live microbe diets, while females benefited from high live microbe diets. All subgroup analysis results are shown in Table 3.

**TABLE 3:** Subgroup analysis.

Subgroup	Low	Medium	High	P for trend	P for interaction
Age					0.071
<60	Reference	0.48 (0.22, 1.04) 0.062	0.02 (0.00, 0.23) 0.004	<0.001	
≥60	Reference	0.65 (0.43, 0.98) 0.041	0.49 (0.27, 0.87) 0.019	0.016	
Gender					0.710
Female	Reference	0.59 (0.33, 1.05) 0.069	0.33 (0.16, 0.68) 0.005	0.004	
Male	Reference	0.52 (0.29, 0.93) 0.030	0.44 (0.13, 1.48) 0.170	0.162	
Education					0.790
Less than high school	Reference	0.52 (0.26, 1.05) 0.067	0.32 (0.05, 2.07) 0.216	0.084	
High school or GED	Reference	0.57 (0.16, 2.06) 0.366	0.23 (0.04, 1.16) 0.072	0.074	
Above high school	Reference	0.66 (0.35, 1.24) 0.184	0.51 (0.27, 0.97) 0.042	0.048	
Hypertension					0.016
No	Reference	0.75 (0.22, 2.55) 0.623	0.09 (0.01, 0.68) 0.023	0.015	
Yes	Reference	0.53 (0.36, 0.78) 0.003	0.55 (0.32, 0.95) 0.034	0.034	
Diabetes					0.891
No	Reference	0.58 (0.33, 1.02) 0.056	0.40 (0.18, 0.87) 0.024	0.024	
Yes	Reference	0.53 (0.29, 0.97) 0.040	0.33 (0.15, 0.71) 0.008	0.004	
eGFR					0.408
<60	Reference	0.61 (0.25, 1.53) 0.271	0.59 (0.22, 1.62) 0.284	0.271	
≥60	Reference	0.58 (0.39, 0.87) 0.012	0.33 (0.16, 0.67) 0.004	0.003	
BMI					0.040
<25	Reference	1.53 (0.60, 3.88) 0.345	0.65 (0.21, 1.99) 0.429	0.306	
≥25	Reference	0.47 (0.35, 0.65) < 0.001	0.36 (0.19, 0.66) 0.003	0.002	
Smoking					0.307
No	Reference	0.79 (0.41, 1.51) 0.443	0.55 (0.25, 1.22) 0.131	0.128	
Yes	Reference	0.45 (0.27, 0.77) 0.006	0.30 (0.13, 0.69) 0.008	0.006	
Alcohol use					0.166
No	Reference	0.35 (0.07, 1.67) 0.173	1.03 (0.25, 4.18) 0.970	0.917	
Yes	Reference	0.57 (0.40, 0.80) 0.003	0.34 (0.20, 0.59) < 0.001	<0.001	

The results of subgroup analysis were adjusted for all covariates except the effect modifier.

### 3.4 Sensitivity analysis

To prevent bias caused by excluding samples with missing covariates, we also performed multiple imputations. The results showed that this correlation remained robust. In addition, we used the AAC8 scoring system to define patients with AAC8 scores greater than or equal to 3 as severe AAC. The results of the correlation analysis also remained stable. All sensitivity analysis data are in the Supplementary material.

## 4 Discussion

To our knowledge, this is the first study examining the association between dietary live microbe intake, assessed through NHANES 24-h recall data, and AAC within a nationally representative sample of US adults. Our findings indicated that compared to the low intake group, the high dietary live microbe intake group was associated with lower AAC scores and lower risk of severe AAC. The observed relationship persisted even after adjustment for relevant confounders, including demographics, bone metabolism markers, kidney function, other laboratory tests, comorbidities, drug use, and energy intake. In the subgroup analysis, the benefit of increased live microbe intake was particularly pronounced among age above 60 years, eGFR ≥ 60 ml⋅ (min × 1.73 m^2^) ^–1^ and those with cardiovascular risk factors like hypertension, smoking, alcohol use, overweight, and diabetes. These findings underscored the potential therapeutic role of dietary live microbes, especially for individuals facing heightened cardiovascular risks, and these deserved further exploration and validation in future research.

Our findings are consistent with previous research, reinforcing the potential benefits of dietary interventions. A dietary intervention randomized controlled trial (RCT) included 90 patients with CVD risk factors who received probiotic alone, lactofermented Annurca apple puree (lfAAP), or unfermented Annurca apple puree (AAP) for 8 weeks according to a 1:1:1 allocation. At the end of the intervention, compared with other groups, the treatment effect of the lfAAP group was most obvious; HDLC increased by 61.8% compared with before intervention, while trimethylamine N-oxide (TMAO) decreased by 63% (28). Moreover, it enhanced the bioavailability of dietary polyphenols in the gut to exert antioxidant and anti-inflammatory cardiovascular benefits (28–30). A recent study found that participants who consumed medium or high levels of dietary live microbes showed better cognitive function compared to those with low intake, particularly for those with certain medical conditions like CVD, diabetes, and hypertension (23). Taking into consideration recent research, the potential health advantages associated with augmenting dietary live microbe intake, spanning improvements in blood pressure, glucose and lipid metabolism, cognitive function, intestinal nutrient absorption, and its influence on the intestinal flora, may indeed form a fundamental strategy for mitigating the risk of severe AAC (21, 23).

Previous studies have observed changes in the gut flora of VC patients. An observational study investigated differences in gut flora composition among chronic disease patients with varying degrees of aortic arch calcification (AoAC) (31). Individuals with the highest AoAC scores displayed a significant decrease in α-diversity along with a heightened prevalence of *Clostridia* species; those with lower AoAC scores exhibited a more favorable microbial profile, characterized by a higher abundance of beneficial bacteria such as *Agathobacter* (31). In another study involving 73 hemodialysis patients, notable distinctions in gut flora were discerned across various VC groups (32). *Escherichia coli* exhibited a positive correlation with VC and emerged as the primary contributor to VC progression; conversely, *Ruminococcus*, the bacterium recognized for producing short-chain fatty acids (SCFAs), displayed a negative correlation with VC and had the second most significant impact on VC (32). These SCFAs can promote tissue repair, regulate immunity, and are closely related to VC (33). In the rat vascular calcification model induced by vitamin D3 and nicotine, it was found that *Akkermansia* supplementation can enhance intestinal flora diversity, promote SCFA production, reduce inflammation, and ultimately alleviate VC (9). Consequently, these findings suggest a potential role for the gut flora in the development and progression of AAC.

One plausible explanatory mechanism for the observed association involves the food-gut-health axis (34). Diet can exert selective pressure on the gut flora, determining which microorganisms can colonize, persist, or become extinct in the gastrointestinal tract (35). It therefore plays a key role in shaping the composition and diversity of the gut microbiota (35, 36). Fermented Foods, fresh fruits and vegetables are an important source of probiotics, including *Lactobacillus*, *Bifidobacterium*, and *Escherichia coli* (37, 38). Probiotics exert anti-vascular calcification effects by acting on the intestines and throughout the body (39). First, probiotics play a pivotal role in restoring microbial equilibrium by upholding the integrity of the intestinal epithelial barrier and facilitating rebalancing (40). This maintenance protects the intestinal barrier, reduces the overpopulation of harmful bacteria, and prevents the production and leakage of harmful bacterial by-products into the circulation (41). It can regulate the expression of specific microRNAs and inhibit synthetases to reduce the production of TMAO metabolites (42, 43). Furthermore, the beneficial effects of probiotics extend to regulating the absorptive function of the intestines (44). They aid in the conversion of inorganic zinc into its organic form, thereby promoting its absorption—an action that contributes to the fight against AS and VC (44). Moreover, probiotics can promote the absorption of nutrients such as polyphenols, magnesium, vitamin D, vitamin C, and vitamin E (29, 45, 46). Not only that, it can inhibit the absorption of heavy metals and cholesterol in the intestine, and the heavy metals are thought to promote AS and calcification (47). Additionally, probiotics contribute to the production of substances associated with the prevention of VC, including vitamin K, and SCFAs (33, 48). These findings underscore the diverse and impactful role of probiotics in promoting gut-health and their potential implications for broader systemic wellbeing.

Our focus on habitual dietary microbe intake, rather than probiotic supplements, enhances the translational potential of these findings. A sustained, stable, and long-term dietary regimen significantly influences the composition and functional dynamics of the gut microbiota (49), and the impact of probiotics tends to be relatively transient (50, 51). However, we cannot directly recommend that the general population increase their intake of dietary live micorbes. Because its long-term effects are unknown, and its safety needs to be considered, especially for certain special groups including those with multiple severe infections, immune deficiencies, or gastrointestinal inflammation (52). In addition, the effects of dietary interventions enriched with dietary live microbes may vary by strain. Therefore, it may be important to emphasize tailoring the approach to individual health status and goals. Nonetheless, current evidence suggests that increasing the intake of microbe-rich foods may be a valuable strategy for improving cardiovascular health. It may have practical implications for public health and dietary guidelines. But longitudinal studies and RCTs are necessary.

There are several limitations in this study. First, in this cross-sectional study we observed the association, but causation cannot be established. Second, 24-h dietary recall data may be inaccurate due to recall bias and dietary live microbes can be affected by transportation, storage, and cooking. Third, Sanders’ dietary live microbe classification system may have lower accuracy than direct measurement. However, direct measurement requires a long time and huge expenditure, which limits its application. Fourth, the rough grouping of microbial diets may introduce estimation errors in assessing microbial intake. Fifth, our study is based on the US population, and the generalizability of the results to other populations worldwide may be limited. Sixth, although NHANES covers most of the US population through complex sampling, it does not include the hospitalized population. This results in under-assessment of critically ill patients. Seventh, despite adjusting for confounding factors, there may still be unknown confounders that have not been accounted for. Therefore, our research results should be treated with caution and can not directly guide the diet of the population. Nonetheless, our study provides new evidence for the health benefits of a diet rich in live microbes, and we call on more researchers to conduct further studies on dietary live microbes.

## 5 Conclusion

Our study demonstrated that more intake of dietary live microbes was associated with a reduced AAC score and the risk of severe AAC among United States adults. However, more studies are still needed to validate our findings.

## Data availability statement

Publicly available datasets were analyzed in this study. This data can be found here: https://www.cdc.gov/nchs/nhanes/index.htm.

## Ethics statement

The studies involving humans were approved by the National Center for Health Statistics Ethics Review Board. The studies were conducted in accordance with the local legislation and institutional requirements. Participants provided written informed consent for participation in NHANES. The data were de-identified and all participant data were obtained from publicly available NHANES. Therefore, this study did not require further approval and followed ethical guidelines.

## Author contributions

XH: Conceptualization, Data curation, Formal analysis, Investigation, Writing – original draft, Writing – review and editing. SJ: Writing – original draft, Writing – review and editing, Conceptualization. XZh: Investigation, Writing – review and editing. LS Investigation, Writing – review and editing. XL: Investigation, Writing – review and editing. LL: Investigation, Writing – review and editing. XZu: Investigation, Writing – review and editing. XC: Funding acquisition, Investigation, Supervision, Writing – review and editing.

## Footnotes

1
wwwn.cdc.gov/nchs/nhanes/Default.aspx
