# Supplementary material for: Association of dietary live microbe intake with abdominal aortic calcification in US adults: a cross-sectional study of NHANES 2013–2014
Source: Front Nutr. 2023 Nov 24;10:1267607. doi: 10.3389/fnut.2023.1267607 (PMC10704926; doi:10.3389/fnut.2023.1267607)
Supplement: Supplementary file 1 [file Data_Sheet_1.docx]

**Supplement Table 1**. The results of Association between Dietary Live Microbes and AAC after multiple imputations.

|  | β^a^/OR^b^ (95% CI^c^), P-value | | | |
| --- | --- | --- | --- | --- |
|  | Low | Medium | High | P for trend |
| **AAC^d^ scores** | | | | |
| Model 1^e^ | Reference | -0.04(-0.29, 0.21) 0.733 | -0.47(-0.92, -0.03) 0.039 | 0.039 |
| Model 2^f^ | Reference | -0.27(-0.52, -0.02) 0.037 | -0.63(-1.10, -0.16) 0.018 | 0.014 |
| Model 3^g^ | Reference | -0.26(-0.49, -0.03) 0.030 | -0.51(-0.87, -0.16) 0.008 | 0.008 |
| **severe AAC^d^** | | | | |
| Model 1^e^ | Reference | 0.92(0.74, 1.15) 0.441 | 0.54(0.30, 0.95) 0.034 | 0.018 |
| Model 2^f^ | Reference | 0.62(0.49, 0.79) 0.004 | 0.40(0.22, 0.75) 0.013 | 0.007 |
| Model 3^g^ | Reference | 0.59(0.48, 0.74) <0.001 | 0.41(0.24, 0.70) 0.003 | 0.002 |

**β**^a^: effect sizes;

OR^b^: odds ratio;

95% CI^c^: 95% confidence interval;

AAC^d^: abdominal aortic calcification;

Model 1^e^: adjusted for non covariates;

Model 2^f^: adjusted for age, gender, race, education;

Model 3^g^: further adjusted for body mass index, systolic blood pressure, diastolic blood pressure, smoking, alcohol use, hypertension, diabetes, congestive heart failure, hba1c, total cholesterol, uric acid, estimated glomerular filtration rate, creatine, potassium, calcium, phosphorus, total 25-hydroxyvitamin D, white blood cells, antidiabetic, antihypertensive, antihyperlipidemic and dietary energy.

**Supplement Table 2.** Using AAC8 score greater than or equal to 3 as the diagnostic criteria for severe AAC to analyze the correlation between dietary viable bacteria and AAC.

|  | β^a^/OR^b^ (95% CI^c^), P-value | | | |
| --- | --- | --- | --- | --- |
|  | Low | Medium | High | P for trend |
| **AAC^d^ scores** | | | | |
| Model 1^e^ | Reference | -0.08(-0.19, 0.03) 0.122 | -0.21(-0.35, -0.07) 0.006 | 0.006 |
| Model 2^f^ | Reference | -0.17(-0.25, -0.09) 0.002 | -0.27(-0.41, -0.13) 0.003 | 0.002 |
| Model 3^g^ | Reference | -0.16(-0.25, -0.07) 0.002 | -0.22(-0.33, -0.10) 0.001 | 0.001 |
| **severe AAC^d^** | | | | |
| Model 1^e^ | Reference | 0.82(0.62,1.10) 0.172 | 0.51(0.32,0.80) 0.007 | 0.005 |
| Model 2^f^ | Reference | 0.57(0.42,0.78) 0.004 | 0.39(0.23,0.65) 0.004 | 0.002 |
| Model 3^g^ | Reference | 0.55(0.40,0.76) 0.001 | 0.42(0.28,0.64) <0.001 | <0.001 |

β^a^: effect sizes;

OR^b^: odds ratio;

95% CI^c^: 95% confidence interval;

AAC^d^: abdominal aortic calcification;

Model 1^e^: adjusted for non covariates;

Model 2^f^: adjusted for age, gender, race, education;

Model 3^g^: further adjusted for body mass index, systolic blood pressure, diastolic blood pressure, smoking, alcohol use, hypertension, diabetes, congestive heart failure, hba1c, total cholesterol, uric acid, estimated glomerular filtration rate, creatine, potassium, calcium, phosphorus, total 25-hydroxyvitamin D, white blood cells, antidiabetic, antihypertensive, antihyperlipidemic and dietary energy.
